# Beyond Head and Neck Cancer: The Relationship Between Oral Microbiota and Tumour Development in Distant Organs

**DOI:** 10.3389/fcimb.2019.00232

**Published:** 2019-06-26

**Authors:** Marco Mascitti, Lucrezia Togni, Giuseppe Troiano, Vito Carlo Alberto Caponio, Davide Bartolomeo Gissi, Lucio Montebugnoli, Maurizio Procaccini, Lorenzo Lo Muzio, Andrea Santarelli

**Affiliations:** ^1^Department of Clinical Sciences and Stomatology, Marche Polytechnic University, Ancona, Italy; ^2^Department of Clinical and Experimental Medicine, University of Foggia, Foggia, Italy; ^3^Department of Biomedical and Neuromuscular Sciences, University of Bologna, Bologna, Italy; ^4^Dental Clinic, National Institute of Health and Science of Aging, IRCCS INRCA, Ancona, Italy

**Keywords:** oral microbiota, oral microbiome, pancreatic cancer, gastrointestinal tract cancer, high-throughput sequencing

## Abstract

An altered oral microbiota has been linked with the development of several oral diseases, such as dental caries, periodontal disease, and oral stomatitis. Moreover, poor oral health has been linked to head and neck cancer, particularly oral cancer. In recent years a growing number of studies indicate that oral microbiota could be involved in the development of primary tumours outside of head and neck region. The aim of this article is to review the recent studies based on high-throughput technology to present evidences of a relationship between oral microbiota and “non-head and neck tumours.” Oral dysbiosis seem to be more pronounced in patients with tumours of gastrointestinal tract, in particular oesophageal, gastric, pancreatic, and colorectal cancers, paving the way for developing specific oral microbiota test to allow early cancer detection. Regarding other tumour types, the results are promising but highly preliminary and still debated. Currently, there are several factors that limit the generalization of the results, such as the small sample size, the lack of adequate clinical information about patients, the different sequencing techniques used, and biological sample heterogeneity. Although only at the beginning, the analysis of oral microbiota could be the next step in the evolution of cancer therapy and will help clinicians to develop individualised approaches to cancer prevention and treatment.

## Introduction

The term “oral microbiota” refers to the heterogeneous group of microbial species colonizing all the oral cavity surfaces (Le Bars et al., 2017). In particular, about 700 bacterial species have been identified in oral cavity and, more importantly, 35% of them have not been cultured (Farrell et al., 2012). Indeed, the main limitation of microbial culture is the inability to identify the actual diversity of oral microbiota. To determine bacterial ecosystem as a whole, high-throughput sequencing methods have been developed, representing a leap toward characterization of oral microbial community (Benn et al., 2018). Currently, the most common approach to analyse oral microbiota is genome sequencing, through the analysis of 16S rDNA gene sequences. Indeed, the identification of this conserved DNA sequence has provided a universal system for the identification of bacteria. Recently, results from studies based on genome sequencing have started shedding light on the existence of site-specific microbial patterns that might be considered as “healthy/normal oral microbiota” (García-Castillo et al., 2016). The main obstacle to this definition is the highly variable composition of microbial community due to multiple factors, such as diet, age, and site (Arweiler and Netuschil, 2016). Nevertheless, ecological imbalance of microbial community, called dysbiosis, has been extensively studied both in human and animal models. It is characterised by: loss of beneficial microbes, expansion of pathogenic microbes, and general loss of microbial diversity (Meurman and Uittamo, 2008; Petersen and Round, 2014). It is becoming evident that the dysbiosis can lead to cancer development (Schwabe and Jobin, 2013). Three paradigms were proposed to describe the pathogenetic processes involving the microbiota in cancer development: trigger of chronic inflammation and immune responses that promote tumorigenesis and tumour growth; altered metabolic activity leading to increased production of toxic metabolites; abrogation of virus latency leading to malignancies (Plottel and Blaser, 2011).

An increasing number of studies have shown that oral microbiota plays a role in the development of oral diseases, such as dental caries, periodontal disease, and oral stomatitis (Santarelli et al., 2015; Zhang et al., 2018). Poor oral hygiene and periodontal disease have been linked to oral cancer, and increasing evidences suggest that oral microbiota have a role in oral cancer development (García-Castillo et al., 2016). More broadly, is becoming evident that oral dysbiosis is associated with head and neck cancer development. Head and neck tumours originate from several anatomical sites, each associated with its own microbiota. Therefore, is possible that the crosstalk between microbial populations, combined with known risk factors, could drive head and neck carcinogenesis (Hayes et al., 2018). Oral dysbiosis has been also involved in the pathogenesis of systemic diseases. Indeed, recent studies indicate that oral microbiota seems to be involved in the tumours of distant organs, in particular “non-head and neck tumours” (Table 1; Klimesova et al., 2018).

**Table 1:** Published studies on the relationship between oral microbiota and tumours of distant organs, showing principal results.

**References**	**Cancer**	***n*^**°**^ patients**	**Sequencing technique (instrument) [16S rDNA hypervariable regions]**	**Sample**	**Validated results**	**Main findings**
Chen et al., 2015	EC	87 SCC, 63 dysplasia, 85 controls	Pyrosequencing (Roche 454 FLX Titanium) [V3–V4]	Saliva	No	Overall decreased microbial diversity in cancer patients. Oral microbiota of cancer and healthy subjects clustered in opposite directions, while dysplasia patients were located between the 2 groups.
Peters et al., 2017	EC	81 AC, 25 SCC, 210 controls	Synthesis (Illumina MiSeq) [V4]	Oral rinse	No	Overall microbial diversity of AC and SCC patients did not differ significantly from matched controls. *T. forsythia* and *P. gingivalis* were more abundant in AC and SCC cases, respectively.
Snider et al., 2018	EC	32 Barrett esophagus, 17 control	Synthesis (Illumina HiSeq 2500) [V4]	Oral swab	Yes	A 3-taxon model (*Lautropia, Streptococcus*, and an unspecified genus of the order *Bacteroidales*) discriminated between Barrett and controls (AUC = 0.94). Cancer patients with Barrett showed increased levels of *Enterobacteriaceae*.
Hu et al., 2015	GC	34 GC, 17 controls	Synthesis (Illumina MiSeq) [V2–V4]	Tongue coat	No	Overall diversity of tongue coating microbiota was reduced in “thick tongue” phenotype. Thick tongue coating was associated with gastric cancer.
Sun et al., 2018	GC	37 GC, 13 controls	Synthesis (Illumina MiSeq) [V4]	Saliva, plaque	No	Overall increased microbial diversity in cancer patients. A scoring system based on 11 different genera was used to screen gastric cancer (sensitivity = 97%, false positivity rate = 7.7%).
Wu et al., 2018	GC	57 GC, 80 controls	Pyrosequencing (Roche 454 GS-FLX) [V4]	Tongue coat	No	6 bacterial clusters were identified to distinguish cancer patients from controls. Cancer patients showed less abundant bacterial clusters 1–2 and more abundant bacterial cluster 3–6 (cluster 6 had AUC = 0.76).
Farrell et al., 2012	PC	10 PC, 10 controls	Microarray assay (HOMIM array)	Saliva	Yes	2-biomarker model (*N. elongata* and *S. mitis*) distinguished cancer patients from healthy controls (AUC = 0.90). 2-biomarker model (*G. adiacens* and *S. mitis*) distinguished cancer patients from chronic pancreatitis (AUC = 0.68).
Torres et al., 2015	PC	8 PC, 78 other disease, 22 controls	Synthesis (Illumina MiSeq) [V4]	Saliva	No	Overall microbiota diversity of the three groups was very similar. Significative higher ratio of *Leptotrichia* to *Porphyromonas* was found in cancer patients.
Olson et al., 2017	PC	40 PDAC, 39 IPMN, 58 controls	Synthesis (Illumina MiSeq) [V4–V5]	Saliva	No	Overall microbiota in PDAC and IPMN patients was very similar. PDAC cases showed higher proportion of *Firmicutes* and related taxa.
Fan et al., 2018	PC	361 PC, 371 controls	Pyrosequencing (Roche 454 FLX Titanium) [V3–V4]	Oral rinse	No	*P. gingivalis* and *A. actinomycetemcomitans* were associated with a higher risk of cancer. *Fusobacteria* and *Leptotrichia* were associated with decreased risk of cancer.
Han et al., 2014	CRC	47 CRC, 45 controls	Synthesis (Illumina MiSeq) [V2–V4]	Tongue coat	No	Thick tongue coating was associated with cancer patients and microbiota imbalance on the tongue was related to the changes in tongue coating.
Kato et al., 2016	CRC	68 CRC, 122 controls	Synthesis (Illumina MiSeq) [V3–V4]	Oral rinse	No	Colorectal cancer was associated with increased presence of *Lactobacillus* and *Rothia*. The differences of overall microbiota between smokers and non-smokers were higher in cancer patients.
Russo et al., 2017	CRC	10 CRC, 10 controls	Synthesis (Illumina MiSeq) [V3–V4]	Saliva	No	There were no significant differences in salivary microbiota between cancer patients and controls.
Flemer et al., 2018	CRC	45 CRC, 21 polyps, 25 controls	Synthesis (Illumina MiSeq) [V3–V4]	Oral swab	No	Overall microbiota differs between cancer patients and controls. A specific oral and faecal microbiota test discriminated healthy controls from patients with cancer and polyps (AUC = 0.94 and 0.98, respectively).
Yang et al., 2018	CRC	231 CRC, 461 control	Synthesis (Illumina HiSeq) [V4]	Oral rinse	No	*T. denticola* and *P. intermedia* were associated with increased cancer risk. Furthermore, 11 common and 16 rare taxa were also associated with cancer risk.
Wang et al., 2014	ALL	13 ALL, 12 controls	Pyrosequencing (Roche 454 GS-FLX) [V1–V3]	Plaque	No	ALL patients had reduced overall microbial diversity. Moreover, “leukemia-associated” taxa were detected.
Yan et al., 2015	LC	10 SCC, 10 AC, 10 controls	Synthesis (Illumina HiSeq 2000) [V3–V6]	Saliva	Yes	Overall microbiota differs between cancer patients and healthy controls. 2-biomarker model (*Veillonella* and *Capnocytophaga*) distinguished SCC and AC from controls (AUC = 0.80 and 0.86, respectively).
Lu et al., 2016	HC	35 HC, 25 controls	Synthesis (Illumina MiSeq) [V3–V4]	Tongue coat	No	Increased overall microbial diversity in HC patients. 2 microbial biomarkers (*Oribacterium* and *Fusobacterium*) discriminated between HC patients and controls (AUC = 0.81 and 0.77, respectively).
Wang et al., 2017	BC	55 BC, 21 controls	Synthesis (Illumina MiSeq) [V3, V4]	Oral rinse	No	There were no significant differences in overall oral microbiota between cancer patients and controls.
Galloway-Peña et al., 2017	AML	59 AML	Synthesis (Illumina MiSeq) [V4]	Oral swab	No	AML patients showed high intra-patient temporal instability of oral microbiota diversity. There was a significant relationship between microbiota changes and risk of infection after induction chemotherapy.

*EC, oesophageal cancer; SCC, squamous cell carcinoma; AC, adenocarcinoma; GC, gastric cancer; PC, Pancreatic cancer; CRC, colorectal cancer; ALL, Acute lymphoblastic leukaemia; LC, lung cancer; HC, hepatic cancer; BC, breast cancer; AML, Acute myeloid leukaemia*.

The aim of this article is to review the recent studies based on genome sequencing to present evidences of a relationship between oral microbiota and tumours of distant organs.

## Pancreatic Cancer

Pancreatic cancer (PC) is a malignant tumour with a 5-year survival rate of 2–9% and a median survival time of 6 months (Mcguigan et al., 2018). Although localised PCs have better survival rate, these patients show absence or non-specific clinical manifestations. The diagnostic delay leads to a fatal outcome, with 80% of PCs that are unresectable at the time of presentation (Vincent et al., 2011). Despite the study of thousands of blood biomarkers for early detection of PC, none of them have been validated for clinical use, suggesting a continuous change of proteins expression over the course of disease evolution (Yachida et al., 2010).

Several reports have demonstrated an association between pathogenic microorganisms and PC, suggesting a role as diagnostic biomarkers. Furthermore, is possible that bacterial dysbiosis could contribute to the formation and progression of PC (Wang and Li, 2015). In addition to the well-known risk factors for PC, several studies have reported a positive association between PC and oral health status. In particular, the relationship between periodontal disease and tooth loss and an increased risk of PC was found, suggesting that toxins exposure and increase of systemic inflammation could promote tumour development (Hujoel et al., 2003; Stolzenberg-Solomon et al., 2003; Michaud et al., 2007). These findings led to the first studies on oral microbiota describing the possible link between oral dysbiosis and non-oropharyngeal cancers (Tables 1, 2).

**Table 2:** Comparison of microbial composition at the genus level in cancer patients.

**Cancer**	**Increased microbes (genus)**	**Reduced microbes (genus)**	**References**
EC	*Streptococcus, Veillonella*	*Neisseria, Rothia, Haemophilus*	Chen et al., 2015; Peters et al., 2017; Snider et al., 2018
GC	*Streptococcus, Veillonella*	*Neisseria, Rothia, Leptotrichia*	Sun et al., 2018; Wu et al., 2018
PC	*Streptococcus*	*Neisseria, Haemophilus, Leptotrichia*	Torres et al., 2015; Olson et al., 2017; Fan et al., 2018
CRC	*Rothia, Actinomyces, Lactobacillus*	*Streptococcus, Neisseria, Haemophilus*	Han et al., 2014; Kato et al., 2016; Flemer et al., 2018; Yang et al., 2018
ALL/AML	*Veillonella, Streptococcus*	*Leptotrichia*	Wang et al., 2014; Galloway-Peña et al., 2017
LC	*Veillonella*	*Neisseria*	Yan et al., 2015
HC	*Leptotrichia*	*Streptococcus, Haemophilus*	Lu et al., 2016

*Only main microbial species were reported (for further details, see Supplementary Table 1). Interestingly, expression levels of genus Streptococcus change significantly depending on tumour type. EC, oesophageal cancer; GC, gastric cancer; PC, Pancreatic cancer; CRC, colorectal cancer; ALL/AML, Acute lymphoblastic leukaemia/acute myeloid leukaemia; LC, lung cancer; HC, hepatic cancer*.

In 2012 was conducted the first microbiota profiling of salivary samples of patients affected by PC (Farrell et al., 2012). A marked reduction of *N. elongata* and *S. mitis* and a significant increase of *G. adiacens*, suggesting a link between variations in oral microbiota and PC. In another study, saliva from 108 subjects was evaluated to determine microbiota profiles (Torres et al., 2015). Although the overall microbiota diversity of the groups was similar, a significative higher ratio of *Leptotrichia* to *Porphyromonas* (LP ratio) was found in PC patients. Despite the small sample size, salivary LP ratio was suggested as a simple biomarker for PC. Moreover, this study confirmed previous results showing that patients with high levels of plasma antibodies to *P. gingivalis* were at higher risk of PC (Ahn et al., 2012b; Michaud et al., 2013). Oral microbiota of patients with intraductal papillary mucinous neoplasms (IPMN) and pancreatic ductal adenocarcinoma (PDAC) was recently studied (Olson et al., 2017). The results found similar overall microbiota profile in saliva of PDAC and IPMN patients, but the mean relative proportion of taxa among IPMN patients was between that for PDAC patients and the controls. Recently, Fan et al. conducted the first prospective case-control study with the aim to evaluate microbiota profile in mouthwash samples taken from PC patients and healthy controls (Fan et al., 2018). Although no significant differences in overall microbiota composition were found, *P. gingivalis* and *A. actinomycetemcomitans* were associated with a higher risk of PC, while *Fusobacteria* and *Leptotrichia* were associated with decreased tumour risk.

## Upper Gastrointestinal Tract Cancer

Upper gastrointestinal cancers are characterised by non-specific clinical manifestations, absence of effective treatments, and changing epidemiological trends. Oesophageal cancer is the sixth most common cause of cancer death, with a 5-year survival rate of 15–25%. Histologically, the two main types are oesophageal squamous cell carcinoma (SCC) and adenocarcinoma (AC) (Domper Arnal et al., 2015). Microbiota seems to play a role in oesophageal cancer aetiology, probably through the regulation of the barrier function, the modulation of inflammation, or the direct metabolism of carcinogens (Peters et al., 2017). Furthermore, periodontal disease seems to be associated with increased cancer risk, suggesting a role of oral microbiota (Michaud et al., 2007).

Salivary microbiota of oesophageal SCC, oesophageal dysplasia, and healthy subjects were compared, showing reduction of several genera in cancer patients (Chen et al., 2015). Overall oral microbiota of oesophageal SCC and controls clustered in opposite directions, while dysplasia patients were located between the 2 groups. Oral microbiota profiling was prospectively studied in oesophageal SCC and AC, using pre-diagnostic oral wash samples from cancer and healthy cohorts, but lack of significant diversity between cancer patients and matched controls was found (Peters et al., 2017). Nevertheless, two periodontal pathogens, *T. forsythia* and *P. gingivalis*, were more abundant in oesophageal AC and SCC cases, respectively. A recent work evaluated the feasibility of early detection of Barrett's oesophagus through the assessment of oral microbiota (Snider et al., 2018). A 3-taxon model including *Lautropia, Streptococcus*, and a genus of the order *Bacteroidales* distinguished Barrett's oesophagus from controls with high sensitivity (96.9%) and specificity (88.2%) (Tables 1, 2).

Gastric cancer (GC) is the third most common cause of cancer death, with a 5-year survival rate of 30%. Interestingly, 5-year survival rate in Japan is 70% for early stages of GC, suggesting the feasibility of mass screening programs (Karimi et al., 2014). The well-established role of *H. pylori* in GC development has given rise to the hypothesis that oral microbial dysbiosis could promote GC development (Ahn et al., 2012a). Several epidemiological studies corroborated such hypothesis showing the association between periodontal disease and tooth loss and an increased risk of GC (Stolzenberg-Solomon et al., 2003; Michaud et al., 2017). These findings led to the search for oral microbial biomarkers that allow an early and non-invasive diagnosis of GC (Table 1).

The relationship between tongue coating appearance and tongue microbiota profiling was investigated in GC patients and healthy controls (Hu et al., 2015). Tongue coating was significantly ticker in GC patients and was associated with reduced overall microbial diversity. Another study explored the alterations of tongue coating microbiota in patients with newly diagnosed GC and healthy controls, demonstrating a significative lower ratio of *Bacteroidetes* to *Firmicutes* in GC, especially in non-cardia GC patients (Wu et al., 2018). A recent case-control study was conducted with the aim to investigate the microbial community of both salivary and subgingival plaque in GC patients (Sun et al., 2018). Although saliva and plaque showed two different microbiotas, the overall microbial diversity was significantly increased in GC patients, and a screening system based on 11 different genera demonstrated high sensitivity (97%) (Table 2).

## Lower Gastrointestinal Tract Cancer

Colorectal cancer (CRC) is one of the most diagnosed malignancy and the second most common cause of cancer death (Marley and Nan, 2016). A possible association between gut dysbiosis and CRC have been suggested. Indeed, both the whole colon microbiota and specific pathogens seems to contribute to the formation and progression of CRC (Klimesova et al., 2018). Findings of colonization of CRC tissues by typical oral bacteria raised the hypothesis of a possible involvement of oral microbiota in CRC development. The possible link between oral microbiota and CRC has been suggested by the fact that each day 10^11^ oral bacteria reach colorectal mucosa, influencing local microbiota (Klimesova et al., 2018). Furthermore, several epidemiological investigations suggested an association between periodontal disease and CRC (Hu et al., 2018). Although the interactions between CRC and the oral-gut microbiota axis are poorly understood, several studies have been conducted to investigate the role of oral bacteria as diagnostic biomarkers for CRC (Table 1).

Han et al. evaluated the relationship between tongue coating appearance and microbiota profiling in patients with CRC (Han et al., 2014). The results were similar to those obtained by the same research group in GC (Hu et al., 2015), showing a significant association between “thick tongue” phenotype and overall microbiota imbalance, especially in CRC patients. Another study investigated the association of smoking and CRC history with oral microbiota profile (Kato et al., 2016). The results failed to support earlier observations about the association between *F. nucleatum* and CRC, although increased presence of *Lactobacillus* and *Rothia* genera was found in cancer patients. Furthermore, smoking status was associated with a decrease in *Betaproteobacteria* class and *Veilonellaceae* family, especially in CRC patients. Microbial communities in a small group of CRC patients were investigated by comparing microbiota profiles from saliva, faeces and tissue samples (Russo et al., 2017). Although an altered microbial composition in faecal samples of CRC patients was noted, the differences in oral microbiota between cancer patients and healthy controls were inconsistent. A recent study compared microbiotas from faecal samples and colonic mucosa with oral microbiota in a larger cohort of patients (45 with CRC, 21 with colorectal polyps, and 25 healthy controls) (Flemer et al., 2018). Overall microbiota differs significantly between CRC patients and controls, suggesting that the colonic establishment of a putatively pathogenic oral-like community is more frequent in patients with CRC or polyps. Moreover, a specific oral and faecal microbiota test discriminated healthy controls from CRC patients with high sensitivity (76%) and specificity (94%). Recently, a large prospective case-control study investigated the association of specific periodontal pathogens and the overall oral microbiota with subsequent risk of CRC (Yang et al., 2018). The results showed a significant relationship between CRC risk and higher levels of *T. denticola* and *P. intermedia*. Furthermore, 11 common and 16 rare taxa seemed to be associated with cancer risk, especially among African-American and females.

## Other Tumours

The promising results obtained by oral microbiota studies in gastrointestinal cancers led several research groups to investigate the diagnostic/prognostic applications of oral microbiota in other tumours. Although promising, these results are highly preliminary and far less clear than those reported in the aforementioned studies (Tables 1, 2).

Among the blood cancers, leukaemia is frequently associated with oral manifestations, like periodontitis and candidiasis. Therefore, imbalance of commensal oral microflora could be related to higher risk of both oral and systemic infections (Păunică et al., 2009). Oral microbiota profiling was evaluated in paediatric patients affected by acute lymphoblastic leukaemia (Wang et al., 2014). In particular, a genome analysis was conducted on supragingival plaque microbiota in 13 ALL patients, showing a reduced overall microbial diversity and detecting a specific pool of leukaemia-associated taxa. A longitudinal study was conducted on patients with acute myeloid leukaemia, with the aim to evaluate the variability of oral and gut microbiota during induction chemotherapy (Galloway-Peña et al., 2017). The results highlighted a high intra-patient temporal variability of oral microbiota and a correlation with higher risk of infection after induction chemotherapy.

Among solid malignancies, oral microbiota profile was investigated in lung cancer (LC) patients (Yan et al., 2015). Salivary samples from patients with SCC and AC showed overall microbiota difference between cancer patients and healthy controls. Furthermore, scoring system based on 2 genera, *Veillonella* and *Capnocytophaga*, distinguished healthy controls from SCC and AC. Another high-throughput sequencing-based study investigated tongue coating microbiota in cirrhotic patients with hepatic cancer (HC), finding an increased overall microbial diversity (Lu et al., 2016). Furthermore, 2 genera, *Oribacterium* and *Fusobacterium*, discriminated between HC patients and healthy controls. Finally, Wang et al. compared oral and urinary microbiota from 57 patients with breast cancer (BC) and 21 healthy subjects, but lack of significant relationship between oral microbiota and the presence of BC was found (Wang et al., 2017).

## Conclusions

Overall, the results presented here showed a significant shift in oral microbiota composition between cancer patients and healthy controls. For example, *Firmicutes* and *Actinobacteria* were the most predominant phyla in cancer patients, while *Proteobacteria, Fusobacteria*, and *Bacteroides* were significantly more abundant in healthy controls (Table 2). Recently, the concept of resilience has been applied to the oral microbiota, defined as the capacity of this microbial ecosystem to deal with perturbation without shifting to potentially harmful states (Rosier et al., 2018). Strong and persistent perturbations to the oral environments can disrupt resilience, leading to a “regime shift” toward oral dysbiosis and, ultimately, to an increased risk of oral and systemic disease (Marsh, 2003).

Unfortunately, these results are heterogeneous among the studies, especially regarding specific cancer types, and do not allow drawing general conclusions. In particular, oral microbiota alterations seem to be more pronounced in patients with gastrointestinal tumours, paving the way for developing specific tests to allow early detection of cancer (Farrell et al., 2012; Torres et al., 2015; Flemer et al., 2018; Snider et al., 2018). The overlap between the microbiotas of oral cavity and gastrointestinal tract, probably due to colonization of gut mucosa by oral bacteria, could partially explain these results (Flynn et al., 2016).

There are several factors that currently limit the generalization of these results. First of all, the small sample size, mainly due to the high sequencing cost, limits the statistical power of the studies. Furthermore, lack of adequate information about patients (e.g., oral health status, active smoking, and antibiotic use) is a source of confounding factors (Snider et al., 2018). Most studies are conducted cross-sectionally, a design that is unable to infer causality between oral dysbiosis and tumour development. This aspect is particularly important, because the alternative hypothesis is the so-called “reverse causality,” namely that modifications in the normal oral microbiota are the result of tumour progression, secondary to systemic processes (Fan et al., 2018). Indeed, although different pathogenic mechanisms have been proposed, direct evidence is not yet available and the reverse causality cannot be excluded. In particular, oral microbiota could be involved in cancer development, but specific tissue tropism for microbial translocation and molecular mechanisms of microbiota-driven carcinogenesis at distal sites are yet to be proven (Chen et al., 2017).

Regarding methodological aspects, different types of oral samples were evaluated making the comparison of these microbial communities difficult. Bacteria colonize all sites within the oral cavity, forming different habitats, each with different non-overlapping microbial populations (Jia et al., 2018). Therefore, it is important to develop a method that can access all oral sites and the microbial diversity inherent to them (Lim et al., 2017). Saliva is the most commonly studied sample, due to several features that make it ideal for research purposes. Thanks to its role in “ecological stability” of oral cavity, saliva allows to early detect an overall dysbiotic trend of oral microbiota (Rosier et al., 2018; Belibasakis et al., 2019). Although different collection methods can alter salivary microbiota (e.g., muscle movements from spitting action may provide larger microbial coverage compared with passive drooling), this seems to not significantly affect the overall microbial composition (Lim et al., 2017). The studies reported here used different sequencing techniques and analysed different 16S rDNA hypervariable regions, with an increased risk for ambiguous data for some rare bacterial species (Table 1; Verma et al., 2018). Furthermore, sequencing depth of these studies could not be sensitive enough to adequately describe differences in oral microbiota in patients with and without tumour (Teng et al., 2018).

Nevertheless, the evidences linking oral microbiota with different tumours are promising. Furthermore, metatranscriptomic and metabolomic analyses are making a significant contribution to the understanding of the relationship between oral microbiota metabolism and human health (García-Castillo et al., 2016). The demonstration of a direct causality between oral dysbiosis and risk of tumour development could lead to new prevention strategies. This could also lead to a broader definition of oral health, including also the presence of a stable microbial homeostasis. Furthermore, causal link between oral microbiota and cancer could contribute to the search for new biomarkers (e.g., markers of resilience) and new therapeutic anticancer strategies, such as the use of pre- and pro-biotics (Rosier et al., 2018).

Although only at the beginning, oral microbiota analysis could be the next step in the evolution of cancer therapy and will help clinicians to develop tailored prevention and treatment strategies.

## Author Contributions

MM, AS, and LL conceived the literature review. MM, LT, and GT described pancreatic studies. GT, VC, and LT described upper gastrointestinal tract studies. DG, LM, and AS described lower gastrointestinal tract studies. LL, MP, and LM wrote the concluding remarks. All authors discussed and approved the final version of the manuscript.

### Conflict of Interest Statement

The authors declare that the research was conducted in the absence of any commercial or financial relationships that could be construed as a potential conflict of interest.
