# Supplementary material for: Beyond Head and Neck Cancer: The Relationship Between Oral Microbiota and Tumour Development in Distant Organs
Source: Front Cell Infect Microbiol. 2019 Jun 26;9:232. doi: 10.3389/fcimb.2019.00232 (PMC6607058; doi:10.3389/fcimb.2019.00232)
Supplement: Supplementary file 1 [file Table_1.docx]

**Supplementary Table 1.** Published studies on different composition of oral microbiota in cancer patients and healthy controls. EC = oesophageal cancer; SCC = squamous cell carcinoma; AC = adenocarcinoma; GC = gastric cancer; CRC = colorectal cancer; ALL = Acute lymphoblastic leukaemia; LC = lung cancer; PC = Pancreatic cancer; HC = hepatic cancer; BC = breast cancer; AML = Acute myeloid leukaemia.

| **Author** | **Cancer** | **Taxa** | **Increased microbes** | **Decreased microbes** |
| --- | --- | --- | --- | --- |
| (Chen et al., 2015) | EC | Genus | *Prevotella, Streptococcus, Porphyromonas, Capnocytophaga** | *Veillonella, Neisseria, Rothia, Haemophilus, Treponema** |
| (Peters et al., 2017) | EC  (EAC)  EC  (ESCC) | Class  Order  Family  Genus  Species  Genus  Species | *Actinomycetales*  *Bacteroidetes*  *T. forsythia, A. cardiffensis*  *N. weaveri, T. vincentii,*  *P. nanceiensis* | *Betaproteobacteria*  *Neisseriales*  *Neisseriacea*  *Alloprevotella, Oribacterium, Solobacterium, Neisseria*  *C. durum, P. nanceiensis, N. sicca, S. pneumoniae, L. umeaense, O. parvum, S. moorei, N. flavescens*  *Lachnospiraceae*  *A. paraphrophilus* |
| (Snider et al., 2018) | EC | Phylum  Class  Order  Family  Genus | *Firmicutes, Proteobacteria*  *Clostridia*  *Clostridiales, Enterobacteriales*  *Streptococcaceae, Veilonellaceae, Enterobacteriaceae*  *Streptococcus, Veillonella, Cardiobacterium* | *Proteobacteria*  *Betaproteobacteria*  *Bacteroidales, Burkholderiales, Neisseriales, Cardiobacteriales, Acholeplasmatales*  *Corynebacteriaceae, Burkholderiaceae, Cardiobacteriaceae, Neisseriaceae, Comamonadaceae, Acholeplasmataceae*  *Corynebacterium, Porphyromonas, Filifactor, Bulleidia, Cardiobacterium, Lautropia, Neisseria, Campylobacter, Acholeplasma* |
| (Hu et al., 2015) | GC | Phylum  Class  Order  Family  Genus | *Actinobacteria* | *Proteobacteria, Fusobacteria, Bacteroides*  *Bacteroidia, Betaproteobacteria, Gammaproteobacteria, Fusobacteria*  *Fusobacteriales, Bacteroidales*  *Fusobacteriaceae, Porphyromonadaceae*  *Fusobacterium, Porphyromonas* |
| (Sun et al., 2018) | GC | Genus (saliva)  Genus (plaque) | *Prevotella, Aggregatibacter, Megasphaera*  *Veillonella, Aggregatibacter* | *Leptotrichia, Rothia, Campylobacter, Granulicatella*  *Leptotrichia, Capnocytophaga, Tannerella* |
| (Wu et al., 2018) | GC | Phylum  Genus | *Firmicutes*  *Streptococcus, Abiotrophia* | *Bacteroides, SR1*  *Brevundimonas, Rhizobium, Prevotella, Sphingomonas, Neisseria, Oribacterium, Stomatobaculum** |
| (Farrell et al., 2012) | PC | Species | *G. adiacens* | *N. elongata, S. mitis* |
| (Torres et al., 2015) | PC | Genus | *Porphiromonas* | *Leptotrichia* |
| (Olson et al., 2017) | PC | Phylum  Class  Order  Family  Genus  Species | *Firmicutes*  *Bacilli*  *Lactobacillales*  *Streptococcaceae*  *Streptococcus*  *S. thermophilus* | *Proteobacteria*  *Gammaproteobacteria, Betaproteobacteria*  *Pasteurellales, Neisseriales*  *Pasteurellaceae, Neisseriaceae*  *Haemophilus, Neisseria* |
| (Fan et al., 2018) | PC | Phylum  Class  Order  Family  Genus  Species | *Alloprevotella*  *P. gingivalis,*  *A. actinomycetemcomitans* | *Fusobacteria*  *Fusobacteria*  *Fusobacteriales*  *Leptotrichiaceae*  *Leptotrichia* |
| (Han et al., 2014) | CRC | Genus | *Actinomyces, Leptotrichia* | *Haemophilus, Neisseria* |
| (Kato et al., 2016) | CRC | Phylum  Class  Order  Family  Genus | *Actinobacteria*  *Actinobacteria*  *Lactobacillales, Atinomycetales*  *Lactobacillaceae, Micrococcaceae*  *Lactobacillus, Rothia* |  |
| (Russo et al., 2017) | CRC | Phylum  Class  Order  Family  Genus  Species | No differences | No differences |
| (Flemer et al., 2018) | CRC | Genus |  | *Haemophilus, Parvimonas, Prevotella, Alloprevotella, Lachnoanaerobaculum, Neisseria, Streptococcus* |
| (Yang et al., 2018) | CRC | Family  Genus  Species | *Bifidobacteriaceae, Burkholderiaceae*  *Peptococcus, Anaeroglobus, Mitsuokella, Lautropia*  *P. denticola, B. dentium,*  *L. salivarius, E. yurii, N. oralis, T. lecithinolyticum, Species SR1* | *Carnobacteriaceae, Streptococcaceae*  *Streptococcus*  *P. melaninogenica* |
| (Wang et al., 2014) | ALL | Phylum  Class  Order  Family  Genus | *Firmicutes*  *Bacilli*  *Lactobacillales*  *Aerococcaceae*  *Abiotrophia, Granulicatella, Veillonella* | *Fusobacteria*  *Fusobacteria*  *Fusobacteriales*  *Fusobacteriaceae, Comamonadaceae*  *Comamonas, Leptotrichia* |
| (Yan et al., 2015) | LC | Order  Family  Genus | *Flavobacteriales, Burkholderiales, Campylobacterales, Spirochaetales*  *Veillonellaceae*  *Capnocytophaga, Selenomonas, Veillonella* | *Bacteroidales*  *Lachnospiraceae*  *Neisseria* |
| (Lu et al., 2016) | HC | Phylum  Class  Order  Family  Genus | *Fusobacteria, Actinobacteria, SR1*  *Fusobacteria, Actinobacteria, Epsilonproteobacteria, Clostridia*  *Actinomycetales, Coriobacteriales, Fusobacteriales, Clostridiales, Campylobacteriales*  *Fusobacteriaceae, Leptotrichiaceae, Actinomycetaceae, Lachnospiraceae*  *Fusobacterium, Leptotrichia, Actinomyces, Campylobacter* | *Bacteroidetes*  *Bacteroidia, Gammaproteobacteria*  *Bacteroidales, Pasteurellales, Lactobacillales*  *Streptococcaceae, Pasteurellaceae, Pseudomonadaceae*  *Haemophilus, Streptococcus, Pseudomonas* |
| (Wang et al., 2017) | BC | Phylum  Class  Order  Family  Genus  Species | No differences | No differences |
| (Galloway-Pena et al., 2017) | AML | Genus | *Staphylococcus, Streptococcus, Stenotrophomonas* |  |
